# Conditional survival analysis and dynamic survival prediction for intracranial solitary-fibrous tumor/hemangiopericytoma

**DOI:** 10.1007/s00432-024-05629-1

**Published:** 2024-02-28

**Authors:** Dagang Song, Zhihao Yang, Linqiang Cai, Hua Huang, Zhiwei Gu

**Affiliations:** Department of Neurosurgery, Shaoxing Central Hospital, The Central Hospital of Shaoxing University, Shaoxing, Zhejiang China

**Keywords:** Conditional survival, Overall survival, Intracranial solitary-fibrous tumor/hemangiopericytoma, SEER, Nomogram

## Abstract

**Background:**

As the form of World Health Organization Central Nervous System (WHO CNS) tumor classifications is updated, there is a lack of research on outcomes for intracranial combined solitary-fibrous tumor and hemangiopericytoma (SFT/HPC). This study aimed to explore conditional survival (CS) pattern and develop a survival prediction tool for intracranial SFT/HPC patients.

**Methods:**

Data of intracranial SFT/HPC patients was gathered from the Surveillance, Epidemiology, and End Results (SEER) program of the National Cancer Institute. The patients were split into training and validation groups at a 7:3 ratio for our analysis. CS is defined as the likelihood of surviving for a specified period of time (y years), given that the patient has survived x years after initial diagnosis. Then, we used this definition of CS to analyze the intracranial SFT/HPC patients. The least absolute shrinkage and selection operator (LASSO) regression and best subset regression (BSR) were employed to identify predictive factors. The Multivariate Cox regression analysis was applied to establish a novel CS-based nomogram, and a risk stratification system was developed using this model.

**Results:**

From the SEER database, 401 patients who were diagnosed with intracranial SFT/HPC between 2000 and 2019 were identified. Among them, 280 were included in the training group and 121 were included in the internal validation group for analysis. Our study revealed that in intracranial SFT/HPC, 5-year survival rates saw significant improvement ranging from 78% at initial diagnosis to rates of 83%, 87%, 90%, and 95% with each successive year after surviving for 1–4 years. The LASSO regression and BSR identified patient age, tumor behavior, surgery and radiotherapy as predictors of CS-based nomogram development. A risk stratification system was also successfully constructed to facilitate the identification of high-risk patients.

**Conclusion:**

The CS pattern of intracranial SFT/HPC patients was outlined, revealing a notable improvement in 5-year survival rates after an added period of survival. Our newly-established CS-based nomogram and risk stratification system can provide a real-time dynamic survival estimation and facilitate the identification of high-risk patients, allowing clinicians to better guide treatment decision for these patients.

## Introduction

Solitary-fibrous tumor and hemangiopericytoma are a rare type of meningeal tumor that occurs at a rate of 3.8 cases per 10,000,000 individuals per year in the US (Kinslow and Wang 2020; Kinslow et al. 2018). Since the recent update of the World Health Organization Central Nervous System (WHO CNS) tumor classifications, the solitary-fibrous tumor and hemangiopericytoma have been grouped together due to their common NAB2/STAT6 fusion gene, indicating a likely shared genetic origin and potential variability in clinical behaviors (Sung et al. 2016; Lu et al. 2022; Fritchie et al. 2016; Louis et al. 2016). The recent studies had reported that the incidence of this tumor is increasing, and it required specialized medical attention (Lu et al. 2022). As the form of classification is updated, there is a lack of research on prognostic factors and outcomes for combined SFT and HPC tumors, with previous studies often limited by small sample sizes and an inability to validate findings (Swaminathan et al. 2022a; Mena et al. 1991; Rutkowski et al. 2012; Zeng et al. 2017; Wu et al. 2021). Moreover, there is inadequate description regarding the survival pattern of SFT/HPC patients in contemporary time. The survival description of this tumor is often based on a relatively small sample size and is generally targeted at overall survival (OS) (Wu et al. 2021; Swaminathan et al. 2022b). Further investigation is required to determine the conditional survival (CS) of SFT/HPC.

Currently, surgery with or without adjuvant radiotherapy is the standard of care for with SFT/HPC patients (Kinslow et al. 2018, 2023a). However, due to a lack of controlled trials and prospective studies, the use of radiotherapy varies by institutions and remains controversial (Kinslow et al. 2023b; Sonabend et al. 2014; Prado et al. 2012; Combs et al. 2005; Gou et al. 2022). Also, as tumors are infrequent, past investigations have frequently combined tumors situated in the intracranial and spinal areas, leading to significant dissimilarity in the findings. This suggests that further research should be intensified to focus on intracranial lesions in order to gain greater insight into this type of tumor.

In modern medicine, many studies have suggested that utilizing dynamic survival estimation may aid in the development of effective treatment plans and follow-up strategies that could benefit individuals with cancer (Qian et al. 2023; Luo et al. 2023; Meng et al. 2023). However, no survival prediction tool has been developed thus far for patients with intracranial SFT/HPC. Nomograms are currently the most effective method with graphical algorithms for predicting cancer patient outcomes (Gafita et al. 2021; Sharouni et al. 2021; Berardi et al. 2020). Traditional nomogram incorporated individual predictors but failed to account for survival time, rendering them incapable of delivering real-time survival estimations (Meng et al. 2022). At the same time, CS analysis is a statistical tool that can estimate survival rates over time, thus providing real-time updates to convey the changing dynamics of survival rates (Hieke et al. 2015; Zabor et al. 2013; Jung et al. 2018). Hence, we attempted to combine the nomogram with CS to develop a novel dynamic survival prediction tool, aiming to aid in clinical practice.

The Surveillance, Epidemiology, and End Results (SEER) database is a valuable resource for clinical cancer research as it covers approximately 27.8% of the US population, and it has been widely advocated for the rare tumor study (Harlan and Hankey 2003). Therefore, we conducted this population-based cohort analysis to explore survival pattern and develop a survival prediction tool for SFT/HPC patients.

## Methods

### Data source and patient selection

The SEER program is the National Cancer Institute’s (NCI) authoritative source for research on cancer incidence and survival and it covers approximately 27.8% of the United States population. The vital status of collected patients are regularly reviewed on an annual basis, and the database is subject to frequent quality control checks, thereby supplying clinical researchers with high-quality research samples.

We queried the SEER database to identify cases of SFT/HPC (ICD-O-3 code 8815 and 9150) within the brain (ICD O-3 codes C70.1–C72.9). The following exclusion criteria were used: (1) disease not diagnosed between 2000 and 2019; (2) treatment information was unknown; and (3) the follow-up period was missing. The study gathered patient demographic information from the SEER database, encompassing factors such as age at diagnosis, sex, race, marital status, rural/urban residence, and household income. In addition, the data on tumor site, histological type, tumor size and tumor behavior code ICD-O-3 were collected. The study also acquired treatment and survival information for the patients. Our study utilized OS as the endpoint measuring the period between the patient's diagnosis of the tumor and their death. And OS was analyzed via the Kaplan–Meier method (Stel et al. 2011).

### Statistical analysis

The identified SFT/HPC patients were split into a 7:3 ratio for training and validation groups. Descriptive statistics were used to exhibit the characteristics of the patients, tumors, and treatments for the total cohort, as well as for the training and validation groups.

CS(y|x) represents the probability that a patient who has not succumbed to SFT/HPC at a certain time x after diagnosis will survive an additional y years. Calculations of CS were conducted via standard definition of conditional probability(Skuladottir and Olsen 2003):

$${\text{CS}}\left( {{\text{y}}|{\text{x}}} \right) \, = {\text{OS}}\left( {{\text{y}} + {\text{x}}} \right)/{\text{OS}}\left( {\text{x}} \right)$$

OS(x) and OS(y + x) were survival probability calculated by the Kaplan − Meier methods for x- and (x + y)-years. For example, CS(2|3) represents the probability of a patient surviving for an additional 2 years after surviving for 3 years following their initial diagnosis.

Our study utilized two methods for identifying predictors of prognosis: the least absolute shrinkage and selection operator (LASSO) regression with tenfold cross-validation (lambda.min used as the screening criterion) (Tibshirani 1997), and the best subset regression (BSR) with the maximum adjusted R squared used as the screening criterion (Zhang 2016). The interaction of the variables screened by the two methods was taken as the prognostic factors of the final modeling. A multivariate Cox regression analysis was performed to validate the prognostic significance of selected variables and integrate those predictors to develop a novel CS-based nomogram model.

All variables in our nomogram were quantified as points and upon input of prognostic factors, a personalized OS and CS rates was determined through calculation of total risk points. We also used the maximum standardized log-rank statistic to stratify the risk of patients according to their total risk score for optimization of clinical management. The Kaplan–Meier method was utilized to evaluate the difference in OS between risk groups.

The performance of the model was assessed in both training and validation cohorts. Calibration plots were used to evaluate the consistency between predicted probabilities and observed outcomes. Discrimination in medical terminology refers to the capacity of a nomogram to distinguish between patient prognoses using the Harrell C-index as a quantification measure. The time-dependent receiver operating characteristic (ROC) curves with the area under the curves (AUCs) were employed to assess the accuracy of the survival prediction for the final model. Furthermore, the decision curve analysis (DCA) was adopted to gauge the effectiveness of our nomogram as a medical intervention, in terms of net benefit. R (version 4.1.0) was used for statistical analysis in our study and statistical significance was determined by p values less than 0.05 in a two-tailed test.

## Results

### Clinicopathological characteristics

We identified 401 patients with intracranial SFT/HPC from the SEER database, and these patients were assigned randomly to either the training cohort (*n* = 280) or the validation cohort (*n* = 121). The vast majority of the cohort fell within the less than 60 age range (67.1%), identified as white (81.0%), and resided in a metropolitan county at the time of diagnosis (94.0%). There was no discrepancy in prevalence between the genders. The majority of intracranial SFT/HPC were found in the supratentorial region (60.1%) and diagnosed as HPC type (80.3%). 58.6% of tumors documented by SEER were found to be either benign or borderline in nature. In terms of treatment status, the data suggested that a large proportion of patients underwent surgery procedure (94.0%), and 50.9% of patients received radiotherapy. See Table 1 for details.

**Table 1:** Clinicopathologic characteristics of intracranial SFT/HPC patients

Parameters	Total cohort (*N* = 401)	Training cohort (*N* = 280)	Validation cohort (*N* = 121)
Age in years at diagnosis			
< 60	269 (67.1%)	194 (69.3%)	75 (62.0%)
≥ 60	132 (32.9%)	86 (30.7%)	46 (38.0%)
Sex			
Male	202 (50.4%)	142 (50.7%)	60 (49.6%)
Female	199 (49.6%)	138 (49.3%)	61 (50.4%)
Race			
White	325 (81.0%)	221 (78.9%)	104 (86.0%)
Non-white	76 (19.0%)	59 (21.1%)	17 (14.0%)
Marital status			
Single	155 (38.7%)	113 (40.4%)	42 (34.7%)
Married	228 (56.9%)	154 (55.0%)	74 (61.2%)
Unknown	18 (4.5%)	13 (4.6%)	5 (4.1%)
Tumor site			
Supratentorial	241 (60.1%)	167 (59.6%)	74 (61.2%)
Infratentorial	44 (11.0%)	33 (11.8%)	11 (9.1%)
Brain, NOS	116 (28.9%)	80 (28.6%)	36 (29.8%)
Tumor histology			
Solitary-fibrous tumor	79 (19.7%)	54 (19.3%)	25 (20.7%)
Hemangiopericytoma	322 (80.3%)	226 (80.7%)	96 (79.3%)
Tumor behavior			
Benign and borderline	235 (58.6%)	163 (58.2%)	72 (59.5%)
Malignant	166 (41.4%)	117 (41.8%)	49 (40.5%)
Tumor size			
≤ 50 mm/unknown	249 (62.1%)	171 (61.1%)	78 (64.5%)
> 50 mm	152 (37.9%)	109 (38.9%)	43 (35.5%)
Surgery			
No surgery	24 (6.0%)	20 (7.1%)	4 (3.3%)
STR	208 (51.9%)	145 (51.8%)	63 (52.1%)
GTR	169 (42.1%)	115 (41.1%)	54 (44.6%)
Radiotherapy			
No	197 (49.1%)	132 (47.1%)	65 (53.7%)
Yes	204 (50.9%)	148 (52.9%)	56 (46.3%)
Rural–urban			
Non-metropolitan	24 (6.0%)	20 (7.1%)	4 (3.3%)
Metropolitan	377 (94.0%)	260 (92.9%)	117 (96.7%)
Household income			
< 65,000$	167 (41.6%)	120 (42.9%)	47 (38.8%)
≥ 65,000$	234 (58.4%)	160 (57.1%)	74 (61.2%)

SFT/HPC, solitary-fibrous tumor and hemangiopericytoma; NOS, not other specific; STR, subtotal resection; GTR gross total resection

### Conditional survival analysis

**Fig. 1:**
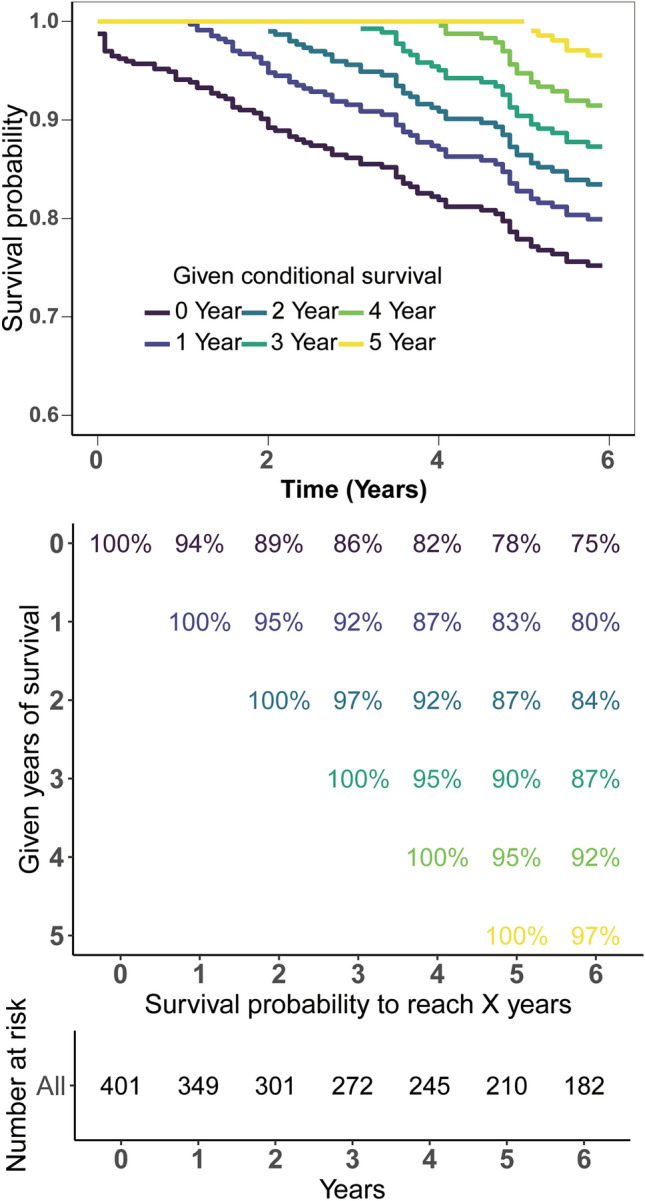
Conditional survival analysis of intracranial SFT/HPC patients. Kaplan–Meier estimates of survival at diagnosis (0 years) and conditional survival based on years already survived after diagnosis (1–4 years). Conditional survival curves (**A**); and updated survival data (**B**) and patient numbers (**C**) adjusted for survived time. SFT/HPC, solitary-fibrous tumor/hemangiopericytoma

The Kaplan–Meier method was used to predict OS probability in SFT/HPC patients and found an OS rate of 86% at the 3-year mark and 78% at 5 years. Through CS analysis, The CS curve for SFT/HPC depicted a rise in 5-year survival rate among these patients for every additional year of survival (Fig. 1). The 5-year survival rate for patients improved gradually from 78% at initial diagnosis to rates of 83%, 87%, 90%, and 95% with each successive year after surviving for 1–4 years, respectively (Fig. 1).

### The CS-based nomogram and risk stratification system construction

**Fig. 2:**
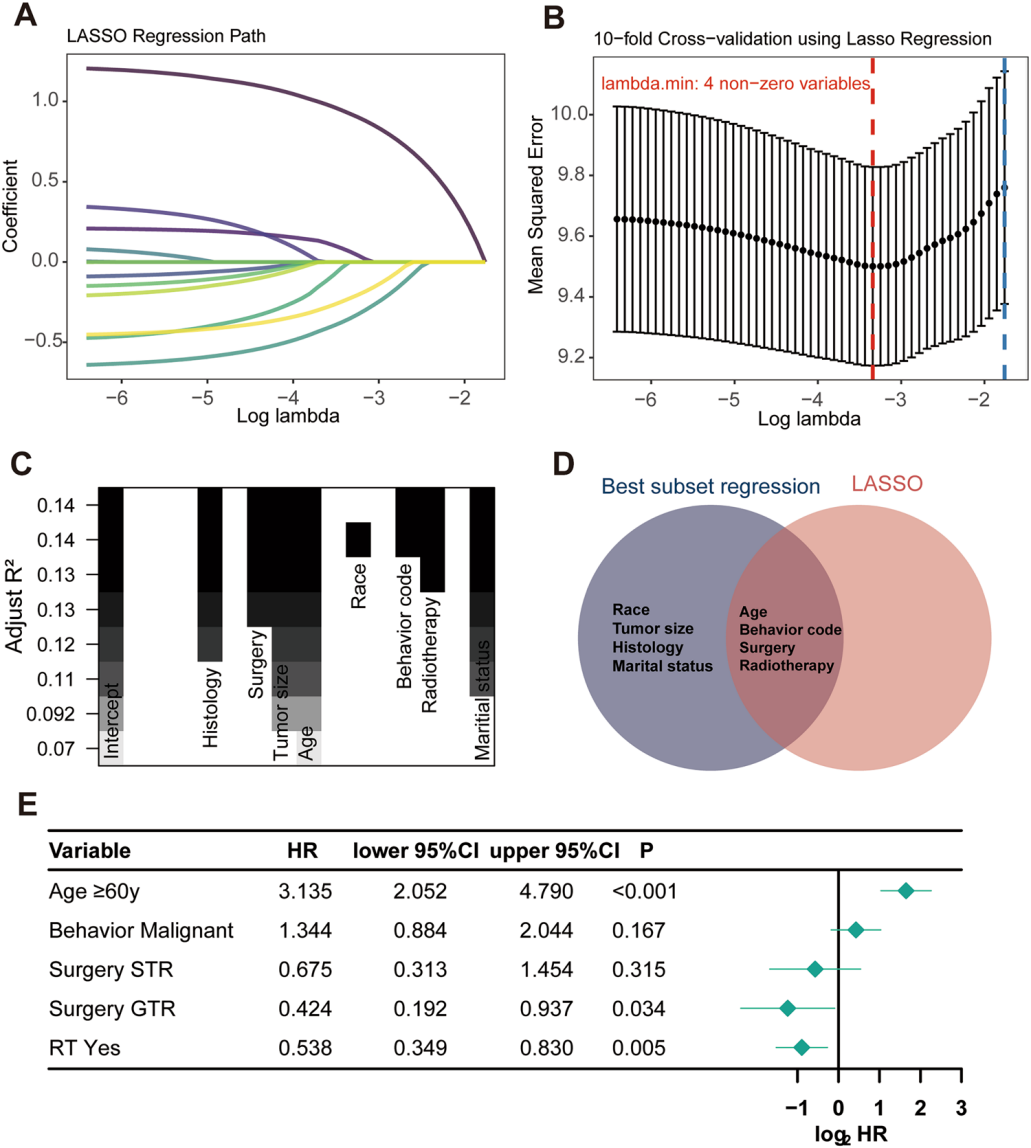
Predictor screening. The least absolute shrinkage and selection operator (LASSO) regression analysis (**A**), and tenfold cross-validation (**B**). Best subset regression (BSR) (**C**). Taking the intersection of the variables selected by the two methods (**D**). Multivariate Cox regression (**E**)

Based on the training cohort, two methods were adopted to screen predictors of SFT/HPC prognosis for prediction model construction: the LASSO regression with tenfold cross-validation identified 4 non-zero variables with lambda.min as screening criteria (Fig. 2A and B), and 8-variable (8/12) combination was selected with the highest adjusted R-squared in BSR analysis (Fig. 2C). Finally, by taking the intersection of the variables selected by the two methods, the subset of 4 variables including age at diagnosis, tumor behavior code, surgery and radiotherapy were screened for nomogram model development (Fig. 2D). The Multivariate Cox regression forest plot further confirmed a significant association between these predictive factors and the prognosis of SFT/HPC (Age, ≥ 60y vs < 60y, HR 3.315, *P* < 0.001; Tumor behavior, Malignant vs Benign and borderline, HR 1.344, *P* = 0.167; Surgery, GTR vs No surgery, *P* = 0.034; Radiotherapy, Yes vs No, *P* = 0.005; Fig. 2E).

**Fig. 3:**
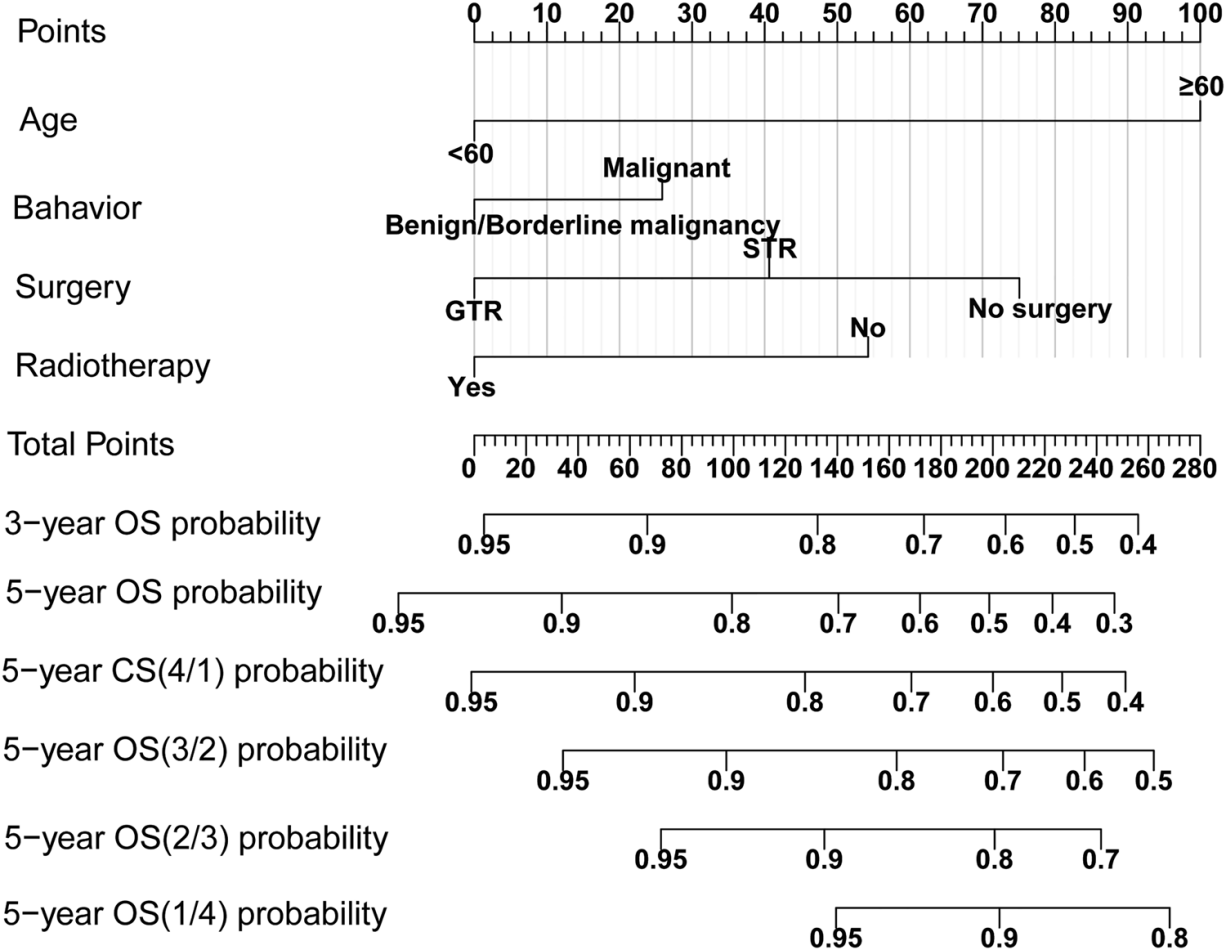
Conditional survival-based nomogram predicting 3- and 5-year overall survival (OS) and 5-year conditional survival (CS) for intracranial SFT/HPC patients. SFT/HPC, solitary-fibrous tumor/hemangiopericytoma; STR, subtotal resection; GTR, gross total resection

**Fig. 4:**
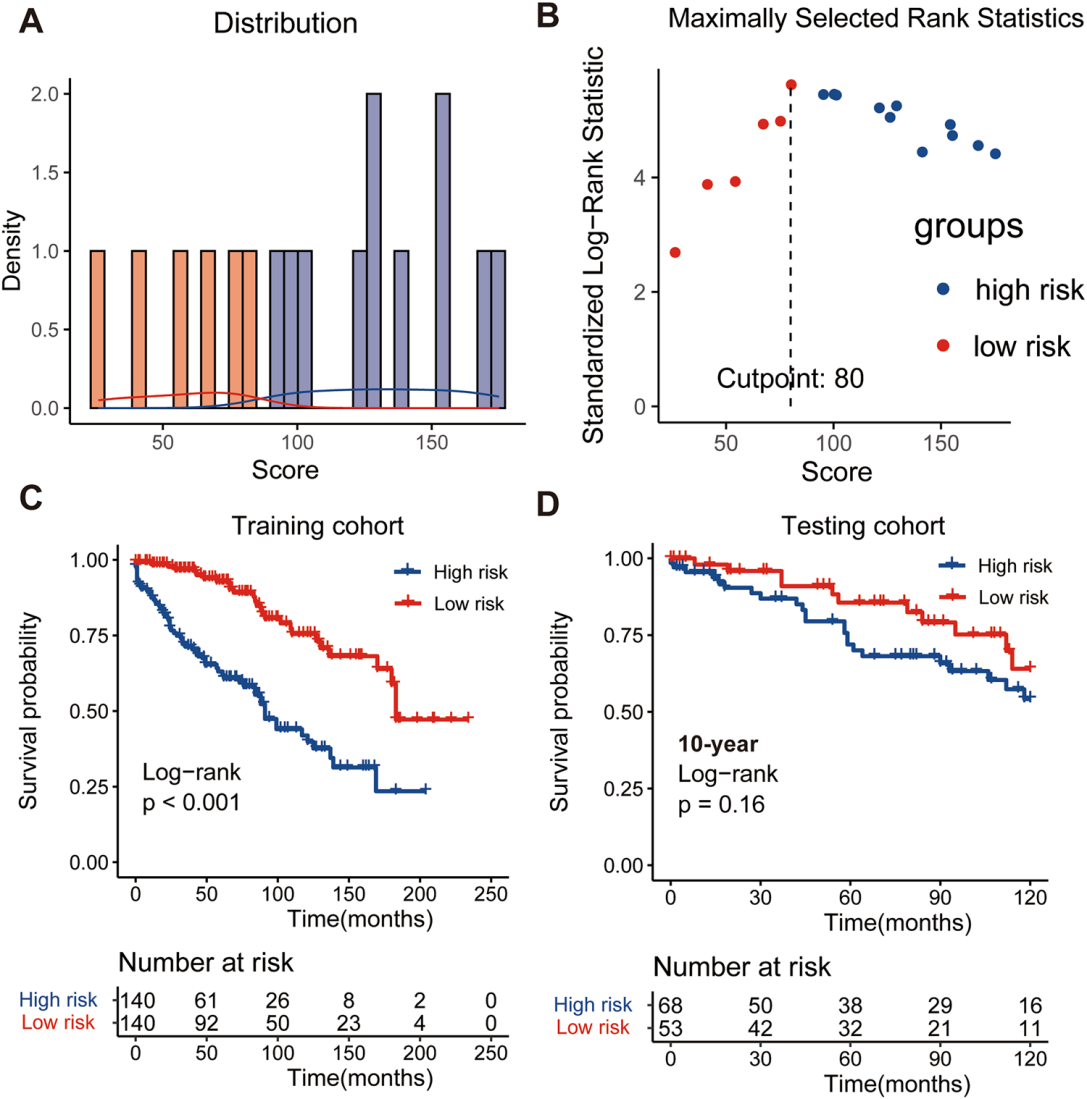
Established a risk stratification system based on the total points of the condition survival nomogram. **A** Distribution of total risk points; **B** the standardized log-rank statistics; **C**, **D** Kaplan–Meier for estimating risk stratification in training and validation cohorts

Then, by combining the CS formula and traditional nomogram model, we successfully integrated above 4 variables to establish a CS-based nomogram model using the Multivariate Cox regression method in training set to estimate 3- and 5-year OS and 5-year CS individually for SFT/HPC patients (Fig. 3). In addition, we utilized the nomogram model to determine the total risk score for each patient and create a system for stratifying their risk. The maximum standardized log-rank statistic indicated that the most appropriate risk cutoff point on survival was at 80, thereby stratifying patients into low- and high-risk groups (Fig. 4A and B). The Kaplan–Meier curves demonstrated that low-risk patients had a significantly better survival advantage than those in the high-risk group in training group (Fig. 4C). In the validation cohort, the risk score also demonstrated a potential association with prognosis, approaching statistical significance with a borderline P value (Fig. 4D).

### The CS-nomogram evaluation and validation

**Fig. 5:**
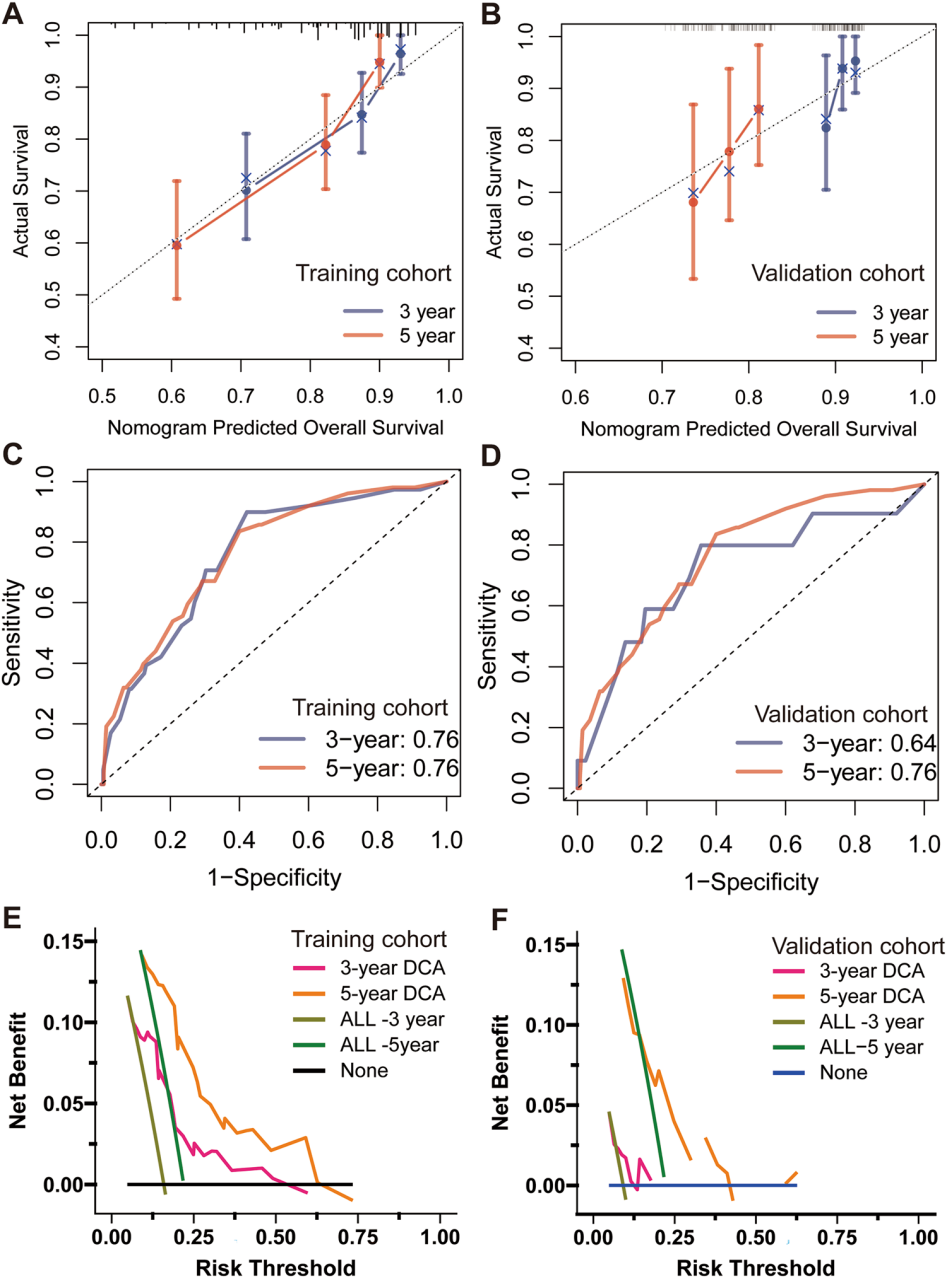
The CS-based nomogram validation. Calibration plots of the nomogram for training (**A**) and validation groups (**B**); Time-dependent area under curve (AUC) curves for training (**C**) and validation groups (**D**). Decision curve analysis (DCA) curves for evaluating clinical usefulness of the nomogram in training (**E**) and validation groups (**F**)

The C-index values of the CS-nomogram were 0.722 and 0.608 in the training and validation groups respectively, indicating that our innovative model displayed good prognostic value. The calibration plots showed this model was well calibrated with good concordance between predicted and observed 3- and 5-year OS probability in both training and validation sets (Fig. 5A and B). ROC analysis revealed that the nomogram exhibited strong discriminatory power in both groups. The 3- and 5-year AUC values were 0.76 in the training group, and 0.64 and 0.76 in the validation group, respectively (Fig. 5C and D). In terms of clinical usefulness, the DCA analysis revealed that the nomograms demonstrated a significant positive net benefit in both training and validation groups (Fig. 5E and F). In DCA curves, the horizontal axis represents the probability threshold and the vertical axis represents the net benefit after subtracting the disadvantage. Both the training and validation cohorts showed improved 3- and 5-year DCA curves for the nomogram, demonstrating its favorable clinical utility.

## Discussion

There have been few studies that have analyzed SFT/HPC together since the update of the WHO guidelines in 2016, likely due to the rarity of these tumors. Those who have conducted mixed tumors in various regions have obtained conflicting findings. Boyett et al. also reported that tumor location was significantly associated with the prognosis of SFT/HPC patients (Boyett et al. 2019). Thus, we conducted a population-based investigation of SFT/HPC with a specific emphasis on intracranial lesions. A CS-based prediction tool was also successfully established for these patients, with the aim of optimizing the clinical management of this disease.

We first described the OS and CS pattern of intracranial SFT/HPC patients and found the 5-year OS of SFT/HPC was 78%. The CS method provides a unique way to assess survival by estimating changes in survival in real-time based on the amount of time already survived. In the recent years, this approach has gained popularity in clinical trials of various cancers, where it is used to analyze changes in the distribution of survival as the disease progresses. Our study revealed that in intracranial SFT/HPC, 5-year survival rates saw significant improvement ranging from 78% at initial diagnosis to rates of 83%, 87%, 90%, and 95% with each successive year after surviving for 1–4 years, respectively. CS prediction analysis provides patients with a visual representation of the dynamic change in their survival probability which can alleviate anxiety in SFT/HPC patients, and facilitate communication and collaboration between doctors and patients.

The alteration of real-time survival estimation depends not solely on the duration of survival, but also on the clinicopathological attributes of the individual (Balachandran et al. 2015; Iasonos et al. 2008). Therefore, we developed a CS-based prognosis prediction tool by combining the traditional model and CS analysis. The LASSO regression analysis and BSR analysis were implemented to screen the variables most significantly related to SFT/HPC’s prognosis and prevent overfitting or underfitting the model. After rigorous selection, a novel CS-based nomogram model was successfully developed integrating four predictive factors including age at diagnosis, tumor behavior code, surgery and radiotherapy. And this model can offer SFT/HPC patients a constantly updated estimation of their chances of survival, with a favorable prediction performance. Furthermore, as a feature of its implementation, this model can be employed for risk stratification by assigning risk scores for SFT/HPC patients. And those patients with high-risk score may require more intensive monitoring and a more proactive course of treatment.

Our multivariate Cox regression analysis also confirmed a significant association between these four predictive factors and the prognosis of SFT/HPC. Our study confirmed prior findings that patients with older age and malignant tumor phenotype were significantly associated with worse outcomes (Kinslow et al. 2018; Lu et al. 2022; Mena et al. 1991; Zeng et al. 2017; Sonabend et al. 2014; Schiariti et al. 2011; Ghia et al. 2013). As the first-line treatment choice for these patients, it is unsurprising that complete surgical removal of tumor was shown to have a significant impact on prognosis, in agreement with previous studies (Kinslow et al. 2018; Lu et al. 2022). It is noteworthy that radiotherapy reception conferred a remarkable survival advantage in our study. Ghose et al. found that survival is improved when complete resection is followed by adjuvant radiation after they systematic reviewed 523 CNS hemangiopericytoma patients (Ghose et al. 2017). Kinslow et al. also supported that gross total resection plus radiotherapy might be optimal in the management CNS SFT/HPC tumors with borderline/malignant feature (Kinslow et al. 2018). However, some researchers reported that the addition of postoperative adjuvant radiation did not seem to confer a survival benefit (Rutkowski et al. 2012, 2010). Owing to the aggressive nature of SFT/HPC, we believe that further radiotherapy may need to be comprehensively evaluated and considered in the high-risk population identified by our nomogram model. Further prospective trials should be conducted to evaluate the effect the radiotherapy.

There are several limitations of our study that require attention. Firstly, due to its retrospective design, there may have been some bias in the selection of data. Prospective cohort studies may be needed to further strengthen the study of this type of tumor. Secondly, the SEER database did not contain some data, including tumor molecular bioinformation, detailed treatment information, and patient complications. Thirdly, we were unable to assess the progression-free survival of these patients due to the limitations of the SEER database. Lastly, our nomogram model needs further externally validation. In the future, it may be necessary to conduct multicenter studies for comprehensive collection of clinicopathological and therapeutic features, in order to determine related prognostic factors and analyze multiple outcome indicators.

## Conclusion

In our study, the CS pattern of intracranial SFT/HPC patients was outlined, revealing a notable improvement in 5-year survival rates after an added period of survival. We also successfully developed the first novel CS-based nomogram model and a risk stratification system for SFT/HPC patients. This model with favorable predictive performance can deliver precise and timely prognostic data, facilitating individualized and economical post-treatment approach and crucial treatment suggestions for patients.

## Data Availability

Publicly available datasets were analyzed in this study. This data can be found here: https://seer.cancer.gov/data/.
